# Autologous Cord Blood in Children with Cerebral Palsy: A Review

**DOI:** 10.3390/ijms20102433

**Published:** 2019-05-16

**Authors:** Dariusz Boruczkowski, Josep-Maria Pujal, Izabela Zdolińska-Malinowska

**Affiliations:** 1 Polski Bank Komórek Macierzystych S.A. (FamiCord Group), Jana Pawła II 29, 00-867 Warsaw, Poland; izabela.zdolinska-malinowska@pbkm.pl; 2 Sevibe Cells, Parc Científic i Tecnològic de la UdG, C/Pic de Peguera No. 11, 17003 Girona, Spain; jmpujal@sevibe.es

**Keywords:** cord blood, stem cells, cellular therapy, cerebral palsy, hypoxia, hypoxic-ischemic encephalopathy, HIE, ischemia, perinatal brain injury, asphyxia

## Abstract

The aim of this narrative review is to report on the current knowledge regarding the clinical use of umbilical cord blood (CB) based on articles from PubMed and clinical trials registered on ClinicalTrials.gov. An increasing amount of evidence suggests that CB may be used for both early diagnostics and treatment of cerebral palsy. The acidity of CB and its biochemical parameters, including dozens of cytokines, growth factors, and other metabolites (such as amino acids, acylcarnitines, phosphatidylcholines, succinate, glycerol, 3-hydroxybutyrate, and O-phosphocholine) are predictors of future neurodevelopment. In addition, several clinical studies confirmed the safety and efficacy of CB administration in both autologous and allogeneic models, including a meta-analysis of five clinical trials involving a total of 328 participants. Currently, nine clinical trials assessing the use of autologous umbilical CB in children diagnosed with hypoxic-ischemic encephalopathy or cerebral palsy are in progress. The total population assessed in these trials exceeds 2500 patients.

## 1. Introduction

The etiology of 70% of cerebral palsy (CP) cases remains unknown. In 20% of children, it may be associated with prematurity, perinatal trauma, or brain hypoxia. Other [specific] causes include infections and defects that arise during fetal life. According to different authors, the frequency of CP is 1.24–3.8/1000 live-born children [1]. The diagnosis of CP is based on a carefully collected medical history, but early diagnosis may be difficult in children with minor alterations. In particular, early diagnosis is important in children with no aggravated perinatal history, as it allows an early start of therapy, especially with stem cells. Cord blood (CB) has been successfully used in CP in both auto- [2,3,4] and allogeneic [5,6] administrations. Animal studies have shown that the timing of stem cell administration is crucial for the effectiveness of CP therapy [7] and suggest that results may also be time-dependent in humans. Therefore, many scientists seek biochemical predictors of CP in CB. The long-term diagnostic value of CB is well-established. In fact, it has been shown that approximately 90 CB proteins are predictors of disorders diagnosed in adults, such as cancer and cardiovascular, neurodegenerative, infectious, and metabolic diseases [8]. A meta-analysis of 51 articles, including over 481,000 children, showed that CB acidity was significantly associated with neonatal mortality (odds ratio [OR] 16.9), hypoxic-ischemic encephalopathy (HIE) (OR 13.8), intraventricular hemorrhage or periventricular leukomalacia (OR 2.9), cerebral palsy (OR 2.3) [9], and the overall incidence of neonatal complications [10]. Other predictive factors for CP are two clusters of 33 cytokines and growth factors [11]. Only one variable–an interleukin (IL)-16 level ≥ 514 pg/mL–predicted a severe outcome, with a sensitivity of 83% and a specificity of 81% [12]. A metabolomic analysis by Walsh et al. showed a significant increase in 29 out of the 148 metabolites (amino acids, acylcarnitines, and phosphatidylcholines) measured in the umbilical CB of infants with either asphyxia or HIE compared to matched healthy controls [13]. A similar study by Ahearne et al. indicated that succinate, glycerol, 3-hydroxybutyrate, and O-phosphocholine levels can also predict the three-year neurodevelopmental outcome in infants with perinatal asphyxia and HIE [14]. CB analysis may also retrospectively explain unexpected clinical outcomes. For instance, a suboptimal concentration of antioxidative micronutrients and heavy metal overload in CB, as well as alterations in DNA methylation in both placenta and CB, are associated with preterm birth [15,16].

## 2. The Scientific Basis for the Use of Umbilical Cord Blood in the Treatment of Cerebral Palsy

### 2.1. Cord Blood Composition and Its Mode of Action

CB contains, among other components, stem cells, anti-inflammatory cytokines, and—to a lesser extent—proinflammatory cytokines. The following cell populations have potential neuroregenerative properties: Umbilical CB-derived mesenchymal stem cells (CB-MSCs); umbilical CB-derived unrestricted somatic stem cells (CB-USSCs); umbilical cord-derived embryonic-like stem cells (CB-CBEs); umbilical CB-derived multipotent progenitor cells (CB-MPCs); umbilical CB-derived endothelial progenitor cells (CB-EPCs); umbilical CB-derived T regulatory cells (CB-Tregs); umbilical vein MSCs (CB-MDSCs); and Wharton’s jelly-derived mesenchymal stem cells (WJ-MSCs) [17,18]. CB cells secrete immunomodulatory and neurotrophic factors that can contribute to central nervous system (CNS) repair [19]. Therefore, the suggested mechanisms for the neuroprotective effect of CB stem cells include neural differentiation, anti-apoptotic and anti-inflammatory actions, the stimulation of angiogenesis, and the production of trophic factors [20,21]. The mode of action depends on the disease [22] and may be multidirectional.

#### 2.1.1. Homing and Neurodifferentiation

The in vitro differentiation of human CB-MSCs into neural cells has been confirmed [23,24] and some studies suggest that these cells can cross the blood-brain barrier like leukocytes [25]. Stem cell homing is a complex process that starts with the migration of the activated stem cells along the concentration gradient and ends with the fulfilment of their regenerative functions [26]. Cell migration begins with the adhesion of the cells to the vascular endothelium at the target tissue and the binding of their homing receptors, followed by extravasation [27]. It is regulated by extracellular growth factors, chemokines, and microRNA [28]. The stromal cell-derived factor 1 (SDF-1) is a chemokine thought to influence human umbilical CB (hUCB) cell migration. SDF-1 is expressed in many tissues, but its expression is increased in the brain after hypoxic-ischemic injury, mainly on the astrocytes and glial cells of the injured hemisphere [29,30,31]. Transplanted hUCB cells expressing the SDF-1 receptor CXCR4 have been observed to migrate to the site of injury within 24 h [32].

After homing, stem cells engage in paracrine signaling to induce target cells to initiate progenitor cell proliferation and tissue repair. Stem cell paracrine activity, their most important therapeutic mechanism, is characterized by the secretion of extracellular vesicles (EVs) and soluble factors. Some of the soluble factors have been proposed for adjuvant stem cell therapy, including granulocyte colony stimulating factor (GCSF), brain-derived neurotrophic factor (BDNF), epidermal growth factor (EGF), and glial cell line-derived neurotrophic factor (GDNF) (Table 1). In particular, BDNF, GDNF, and GCSF are involved in the development, regeneration, and survival of neurons [33,34,35,36,37]. In a rodent model of HIE, MSCs exerted neuroprotective effects by enhancing autophagy through the BDNF/mTOR signaling pathway [38]. GCSF activates STAT proteins and the PI3-K/Akt pathway [39]. Results from non-stroke studies indicate that BDNF affects differentiating oligodendrocytes and improves axonal growth and plasticity by increasing the levels of MBP cells and MBP protein and decreasing Nogo-A levels [34]. In vitro studies show that GPE, the N-terminal tripeptide of the insulin-like growth factor 1 (IGF-1), stimulates neural stem cell proliferation and migration by activating the extracellular signal-regulated kinase (ERK) and PI3K-Akt pathways [40]. The Akt pathway is activated by the endogenous recombinant prion protein [41] that is mainly expressed in the central and peripheral nervous system [42]. 

#### 2.1.2. Extracellular Vesicles

In recent years, the interest of scientists in the role of extracellular vesicles (EVs)—micro- or nanoparticles carrying mRNA, microRNA, and proteins [49]—has grown. EVs were effective in the treatment of animal models of liver diseases [50,51,52], myocardial infarction [53], myocardial hypoxia [54], ischemic-hypoxic kidney injury [55,56], inflammation-induced preterm brain injury [57], traumatic brain injury [58], stroke [59], Parkinson’s disease [60], Alzheimer’s disease [61], and autism [62] (Table 2). They easily cross the blood-brain barrier [63] and accumulate in pathologically relevant brain regions through an inflammation-driven mechanism [64]. MSC-EV administration improves brain function and inflammation-induced neuronal degeneration, inhibits intracerebral inflammation, reduces microglial proliferation, prevents reactive astrocyte proliferation, and improves spatial learning impairments [62]. Short-term myelin deficiency and long-term white matter microstructural abnormalities ameliorated after administration of MSC-EVs [65]. Recently, Lin et al. developed a platform for the neural differentiation of induced pluripotent stem cells (iPSCs), which consisted of polybutylcyanoacrylate nanoparticles with either surface-adsorbed BDNF or encapsulated BDNF. Both methods effectively induced neural differentiation [66]. 

#### 2.1.3. Tunnelling

A tunnelling phenomenon has been described in MSC populations, involving the exchange of molecules and organelles such as calcium ions, prions, viral and bacterial pathogens, small lysosomes and mitochondria [67]. However, whether it is relevant in CP is unknown. 

### 2.2. Efficacy of MSC Administration in Animal Models of Hypoxia

The effectiveness of CB in CP treatment has been repeatedly tested in animal models. In 2006, Meier et al. intraperitoneally administered CB mononuclear cells to rats that had been previously subjected to ischemia by ligation of the carotid artery and showed symptoms of contralateral spastic paralysis. Most of the neurological symptoms were alleviated in the examined animals [68]. In another study, also conducted in a rat model of neonatal hypoxia/ischemia, intravenously administered CB cells transiently increased the activity of microglia in the striatum and had a protective impact on mature neurons of the new cortex, which significantly improved the motor test results of the study group. The results persisted three weeks after the infusion, although in most cases, the presence of the administered cells was not detected in the CNS [69]. Zhang et al. [70] transplanted labeled UC-MSCs and CB mononuclear cells (MNCs) into rats with hypoxia-ischemia (HI)-induced brain damage. They reported reduced astrogliosis, prevention of neuronal loss in the striatum, and a marked improvement in functional brain outcomes after a 28-day recovery period. In addition, treatment with CB-MNCs increased the proportion of mature oligodendrocytes and improved myelination in the cortex. Similar neuroprotective results were obtained by Li et al. in a preterm sheep model of white matter injury. They reported that infusions of UCB-derived MSCs administered after hypoxia-ischemia in the preterm brain reduced white matter injury by inhibiting microglial activation and the release of TNFα, promoting macrophage migration, and accelerating self-repair [71]. In neonatal rats with intraventricular hemorrhage, hUCB-MSCs attenuated neuronal loss and promoted neurogenesis in the hippocampus, which is essential for learning, memory, and cognitive functions. These stem cells also contributed to the restoration of impaired synaptic circuits in the hippocampus, leading to the improvement of neurocognitive functions in these animals through BDNF-TrκB-CREB signaling axis activation [72]. This may explain the improvement in these skills observed in children with CP after WJ-MSC administration [73].

The immunomodulatory properties of T regulatory cells (Tregs) and monocyte-derived suppressor cells (MDSCs) was confirmed by McDonald et al. in the mice model [74]. In this study, infiltration of CD4+ T cells into the injured cerebral hemisphere was significantly reduced by all hUCB cell types evaluated. However, Tregs and EPCs additionally reduced motor deficits, CD4+ T cell infiltration into the brain, and microglial activation. EPCs also significantly reduced cortical cell death and the peripheral Th1-mediated proinflammatory shift, and they returned CD4+ T cell infiltration to sham levels. In a sheep model, human CB reduced white matter cell death and inflammation and recovered total and mature oligodendrocytes [75]. In a rat model, human CB reduced astrogliosis, prevented neuronal cell loss in the striatum, and improved functional brain outcomes. Additionally, CB mononuclear cells increased the proportion of mature oligodendrocytes and improved myelination in the cortex [70].

The paracrine interaction mechanism was confirmed by the preclinical study conducted by Drobyshevsky et al., in which intravenous administration of a large CB dose changed the natural course of hypoxic-ischemic damage in the tested animals. Magnetic resonance imaging (MRI) examination of the CNS showed little penetration of labeled cells, which may suggest a paracrine effect [76]. CB can also be effective in the treatment of neurological deficits caused by intraventricular bleeding [77] or traumatic CNS injury [78]. 

### 2.3. The Influence of Low Oxygen Concentration on Stem Cells

Zhao et al. provided interesting findings concerning the use of autologous CB after hypoxia. They found that when CB CD34+ cells were co-cultured with WJ-MSCs, a physiological low oxygen concentration (1% O_2_) was better for the maintenance of stem cell traits than normal oxygen conditions [79]. The cultures exposed to low oxygen concentration had higher percentages of CD34^+^ Lin^−^ cells and long-term culture-initiating cells. Low oxygen resulted in an increased secretion of vascular endothelial growth factor and a decreased secretion of interleukin (IL)-6, IL-7, stem cell factor, and thrombopoietin by WJ-MSCs. Low oxygen also resulted in the activation of stem cell signaling pathways in the CB stem/progenitor cells. Thus, oxygen concentration may be a tool with important clinical implications. Zhang et al. [80] showed that BM-MSCs cultured at a low (1%) oxygen concentration also displayed greater proliferative potential and reduced apoptosis. The administration of hypoxia-preconditioned MSCs decreased ischemia/reperfusion injury by attenuating inflammatory responses associated with the generation of reactive oxygen species [81]. The influence of oxygen concentration on stem cell properties was recently reviewed by Mas-Bargues et al. [82].

## 3. Clinical Trials Assessing the Use of Autologous Umbilical Cord Blood in the Treatment of Cerebral Palsy

Autologous CB has been used worldwide in transplantology [83,84], hematology [85], cardiac surgery [86], and endocrinology [87,88]. Among other neurological diseases, auto-CB has been used in Duchenne muscular dystrophy [89,90], cerebral palsy [91], and autism spectrum disorder [92,93]. Current studies are exploring the use of umbilical CB in the treatment of HIE (Table 3), congenital hydrocephalus, traumatic brain injury, ischemic stroke, global developmental delay, and ataxia. Autologous peripheral blood was confirmed to be safe and effective in children with cerebral palsy in a double-blind, placebo-controlled trial in 2017 [33].

### 3.1. Early Phase Clinical Studies

Cotten et al. tested the feasibility and safety of providing non-cryopreserved autologous volume- and red blood cell-reduced umbilical CB cells to neonates with HIE and concluded that this procedure is possible, safe, and should be verified in clinical trials [94]. After administration of the same cell fraction, Yang et al. [95] and Kotowski et al. [96] obtained the same results in preterm newborns and very premature neonates, respectively. Additionally, Kotowski confirmed a significant increase of 22 plasma proteins (e.g., insulin-like growth factors, stem cell factor, epidermal growth factors) in recipients of autologous umbilical CB and a decreased percentage of intraventricular hemorrhages in the treated group (40% vs. 86.7%).

In a study by Lee et al. [97], autologous umbilical CB was administered intravenously to 20 patients diagnosed with CP at the age of 2–10 years [mean 55 months]. An average of 5.5 ± 3.8 × 10^7^ [0.6–15.65] total nucleated cells (TNC)/kg of body weight was given. Three patients experienced transient nausea and hemoglobinuria, and the next two patients had hemoglobinuria and urticaria, which disappeared after antihistamine treatment and intravenous hydration. The motor skills and cognitive functions of the patients were evaluated after six months. It was estimated that there was a noticeable improvement in 14 out of 20 patients and that five patients were significantly better than would be expected after standard care for CP. Patients with quadriplegia responded to treatment the worst. MRI track-density imaging (MRI-TDI) and single photon emission computed tomography (SPECT) were also performed, and the results seemed to correlate partially with clinical improvement. 

In 2015, a research group at Duke University [98] announced the first results from a randomized, double-blind, crossover trial on CP treatment with autologous CB. The study involved 63 patients with a median age of two years. The target TNC doses were 1–5 × 10^7^/kg, the final TNC median dose was 2 × 10^7^/kg [0.4–5], the median CD34+ cell dose was 0.5 × 10^5^ /kg [0.05–4]. After the infusion, patients were monitored for 2–4 h. One patient had urticaria and mild fever. The motor assessment of patients one year after administration did not show statistically significant differences between the study and control groups, but a significant difference was found between the group that received infusions with TNC doses >2.5 × 10^7^/kg and the group that received lower doses. The group of patients treated with higher cell doses experienced an improvement in motor skills. 

The same research group published the results of an MRI study on 17 children who took part in the clinical trial described above and experienced a clinical improvement. A strong correlation was found between the increase of white matter integrity and the functional improvement of the patients [99]. In 2017, the same research team published the results of a double-blind, placebo-controlled study on the effects of a single intravenous infusion containing 1–5 × 10^7^ TNC/kg from autologous umbilical CB in children with CP up to six years of age [91]. Motor skills and brain MRI studies were performed at the beginning of the study and one and two years after treatment. Sixty-three children (median age of 2.1 years) were randomly assigned to the treatment group (*n* = 32) or to the placebo group (*n* = 31) at the beginning of the study. Although there was no significant difference in the results between the treatment and placebo groups after one year, a therapeutic effect that was dependent on the number of cells administered to the patients was identified. One year after autologous treatment, the children who received a dose greater than 2 × 10^7^ cells/kg showed a significantly greater improvement on several scales. The results of this study suggest that a properly administered infusion of autologous umbilical CB improves brain function and some motor functions in young children with CP.

Xie et al. reported that in adult patients with HIE, human umbilical cord (hUC)-MSC transplantation was safe and resulted in a significant improvement regarding recovery of neurological function, cognition ability, emotional reaction, and extrapyramidal function [100]. The results were evaluated using several clinical assessment scales, and no significant adverse events were reported during the 180-day follow-up period. 

Huang et al. [101] conducted a randomized, placebo-controlled trial on the use of MSCs from human umbilical CB (hCB-MSC) in 54 children with CP. It was a two-arm study in which, along with basic rehabilitation, one arm received intravenous hCB-MSC infusions at a fixed dose of 5 × 10^7^ cells/kg, and the other received 0.9% normal saline. The endpoints were assessed during the study and during 24 months of follow-up and were the following: Gross Motor Function Measure (GMFM-88), a comprehensive functional assessment (CFA), lab tests, electroencephalogram (EEG), routine MRI, and adverse events. The changes in the total proportion of GMFM-88 and the total CFA scores in the hCB-MSC infusion group were significantly higher than those in the control group three, six, 12, and 24 months after administration. Patients with slowing EEG background rhythms at baseline had less diffused slow waves after hCB-MSC administration. Improvements in cerebral structures as observed by MRI were rare. No serious adverse events were noticed. The authors concluded that hCB-MSC infusion with basic rehabilitation was safe and effective at improving gross motor and comprehensive functions in children with CP.

Bae et al. compared the intravenous application of autologous CB with that of allogeneic CB with a four out of six human leukocyte antigen (HLA) match [102]. In this study, both the safety and efficacy of umbilical CB administration were evaluated. The study group consisted of seven patients with a mean age of 38 weeks. Three patients received allogeneic cells and four received autologous cells. The patients also received erythropoietin 12 h prior to CB administration. The patients in the allogeneic group [*n* = 3] received cyclosporine at a dose of 15 mg/kg before transplantation and for six days after transplantation. For the next three weeks, they received a reduced dose of 10 mg/kg. The group of patients who received allogeneic CB had significantly lower levels of proinflammatory cytokines, such as IL-1beta, tumor necrosis factor-beta, IL-6, and RANTES. In the autologous group, the level of proinflammatory cytokines significantly increased after transplantation. Only the allogeneic group experienced a significant improvement in gross motor performance and social skills. The study was conducted only on seven patients, and the average birth age of the examined children in both groups was significantly different, which could influence the results. To date, no larger study has been published. 

### 3.2. Safety

In cases of intravenous CB infusion in children with CP, fever, nausea, urticaria, hemoglobinuria, increase in blood pressure, and transient decreases in saturation were observed. During the infusion of a blood product, there may be early and late complications. Early complications include nonhemolytic transfusion reactions (such as chills and fever), urticaria, anaphylactic shock, septic shock caused by bacterial infection of the CB unit, transfusion-related acute lung injury, air embolism, acute pain during transfusion, and hypocalcemia associated with transfusion of a CB unit containing citrate. Late complications include, first, transfusion-associated graft versus host disease. In addition, a single transfusion containing an excessive amount of cryoprotective agent can cause dimethyl sulfoxide poisoning (e.g., in the form of neurotoxicity). Infection transmission from non-tested or non-diagnosed infectious agents (e.g., prions) can also occur. However, the risk of such infection is reduced by having access to the mother’s accurate medical history before collecting the CB.

A meta-analysis regarding the safety and efficacy of cellular therapies [103] showed that serious adverse events occurred rarely, only in four out of 135 people in the study groups and in three out of 139 people in the control groups. This meta-analysis covered clinical trials related to umbilical CB and other cells, such as olfactory and nerve cells and nerve cell progenitors.

In 2015, Feng et al. [104] published a retrospective study regarding the safety of the intravenous/intrathecal therapeutic administration of stem cells derived from CB. Forty-seven patients with CP with a median age of 5.85 ± 6.12 years (1–29 years) received an average of 2–3 × 10^7^ CB cells. The number of applications was determined depending on whether side effects occurred, with a minimum of four infusions and a maximum of eight infusions, and the interval between infusions was three to five days. No serious adverse events occurred, although mild ones were observed quite often (in 26 patients) and were mostly fever (42.6%) or vomiting (21.2%). Intrathecal infusion and the ages at the initiation of treatment (≤10 years old) were associated with increased risk of adverse events. It could be associated with the relatively large dose of cells given in relation to the patients’ weight. All adverse events were treated with symptomatic treatment. The follow-up period lasted six months. The treatment procedure was stated as safe, although no immunosuppressive treatment was used and there was no full HLA match between recipient and donor.

Sun et al. [105] demonstrated the safety of multiple infusions of intravenous autologous umbilical CB in children with congenital hydrocephalus. In a study lasting more than 18 years, 76 patients received 143 infusions of autologous CB, including 45 patients who received two infusions, 18 patients who received three, and four patients who received four. There were no infusion-related adverse events. The median age of the treated children was two years (six days–4.5 years). Five CB units had a positive result for bacterial infection after thawing (an earlier result was negative), which were available 24–72 h after the infusion, but the patients did not present clinical symptoms and were not treated with antibiotics. The patients were premedicated with paracetamol (10 mg/kg) and diphenhydramine (0.5 mg/kg) administered orally and methylprednisolone (0.5 mg/kg) administered intravenously. An average of 1.9 × 10^7^ TNC/kg (0.1–13.3 × 10^7^ TNC/kg) and 0.5 × 10^5^ (0–6.4 × 10^5^) CD34+ cells/kg were administered.

## 4. Past and Future Perspectives

Almost 10 years ago, Hadar Arien-Zakay et al. [106] summarized 20 years of CB use, highlighting several issues for future studies. These issues included identifying the preferred cell population, investigating the efficacy of a single CB unit administration for ischemic brain therapy and the possibility of the in vitro expansion of CB-derived cells before transplantation, measuring the significance of the biological differences intrinsic to individual units of CB on the reproducibility of results, and identifying the therapeutic time window. The studies conducted in the last decade have not answered all these questions. Jin [107] showed that UCB-MSCs had the highest rate of cell proliferation and clonality and significantly lowered markers of senescence, compared with bone marrow (BM) and adipose tissue-derived stem cells. However, these results were observed in animals and have not been confirmed in clinical studies, despite arguments confirming their predominance [108]. Some studies have confirmed the superiority of UC-MSCs over BM in terms of secretome [109] and migration speed [110]. An evaluation of the expression of 106 cytokines between UCB-derived and UC-derived MSCs showed differences in the excretion of the following: TSP-1, TSG-14, TIMP-1, IL-8, IL-6, CXCL1, GIF, and IGFBP3. The expression of CCL2 in UC-MSCs was significantly higher than in CB-MSCs, whereas the secretion of IGFBP1 and IGFBP2 by CB-MSCs was higher than by UC-MSCs [111]. This suggests that UC-MSCs and CB-MSCs differ in their functional potentials, which must be carefully considered before clinical treatment. Nevertheless, a recent study in an equine model showed that CB-MSCs have a greater differentiation potential and similar immunosuppressive potential as WJ-MSCs [112]. Data for human cells are still unavailable. On the other hand, DNA methylation in CB and UC differs in human neonates [113]. Therefore, the identification of the best cell population is still an unsolved issue, as is the question regarding the most effective fractions of UCB.

The efficacy of a single CB unit administration was confirmed in children under 10 [97]. A technique of the ex vivo expansion of CB-derived stem cells has been developed, and its clinical efficacy has been positively verified, despite initial problems [114,115,116,117,118]. However, the cells expanded ex vivo undergo changes in genetic expression, which is an issue that requires further studies [119]. Additionally, the parameters of CB units may be ameliorated by improving the CB thawing procedure [120], using DMSO dextrose instead of DMSO [121], and adding new substances before cryopreservation, such as quercetin [122].

The time window for successful therapy has not been established. In a study assessing the efficacy of G-CSF with or without autologous peripheral blood stem cells in children with CP, responders and non-responders did not differ in age, but they were preschoolers (3.2 ± 1.5, 4.7 ± 1.9, *p* = 0.968) [33]. In adults with ischemic stroke, the infusion of a single allogeneic CB unit was enough to obtain a sustained therapeutic effect [123].

In our opinion, besides the issues mentioned above, the most interesting questions for future studies concern the interactions between different types of stem cells and the clinical efficacy of cell therapies combined with substances approved for other indications. The anti-senescent properties of some of these substances may also be a promising strategy to increase the number of passages in stem cell cultures. For instance, although polyunsaturated fatty acids have no influence on CB cytokines, the impact of other CB parameters and clinical outcomes after therapeutic applications of CB is unknown [124]. Resveratrol induced the differentiation of hUC-MSC into neuron-like cells [125], improved hUC-MSC repair in cisplatin-induced acute kidney injury [126], prevented cellular damages induced by monocrotophos via the PI3K signaling pathway in human CB-MSCs [127], and promoted hUC-MSC engraftment and neural repair in a mouse model of Alzheimer’s disease [128]. Quercetin has multiple effects (Table 4). It enhanced the differentiation of BM-MSCs into bone cells in rat [129] and mice models [130]. In vitro, quercetin promoted the differentiation of BM-MSCs into β-cells with increased insulin secretion [131]. In a rat model of spinal injury, a combination therapy including quercetin and hUC-MSCs improved the neurological functional recovery, increased axonal preservation, promoted macrophage polarization, decreased the size of the cystic cavity, reduced the proinflammatory cytokines, and increased anti-inflammatory cytokines compared with hUC-MSCs alone [132]. In human BM cell cultures, quercetin inhibited osteogenetic differentiation and promoted adipogenesis [133]. On the other hand, it stimulated hepatic differentiation and increased cell viability and resistance to oxidative stress [134]. The use of quercetin in rat BM-MSC cultures improved cell proliferation and angiogenic factor secretion in a dose-dependent manner [135]. Quercetin induced cell death in cultures of undifferentiated human pluripotent stem cells (hPSCs) but not human dermal fibroblasts, suggesting it may decrease the risk of teratoma formation during hPSC-based cell therapy [136]. It also exhibited genotoxic effects in human hematopoietic stem and progenitor cells when applied continuously and at high concentrations [137]. Baral et al. investigated the effects of quercetin on neurodifferentiation and stem cell migration, but their results were inconclusive. Although quercetin increased differentiation and migration, it also decreased cell viability [138]. Apigenin increased the expression of neuronal markers and the number of neural progenitor cells obtained from human embryonic stem cells and human iPSCs [139]. Luteolin affected the pro-inflammatory secretion activity of human mast cells [140]. Apigenin and luteolin promoted human hematopoietic stem cell differentiation [141] and were confirmed safe for developing vertebrates in terms of neurobehavior [142].

Studies investigating the mutual interactions between stem cell subpopulations are also in their infancy. It has been shown that CB hematopoietic progenitors influence the expression of genes encoding for adhesion molecules and molecules of the connective tissue matrix [143]. Its remodeling enzymes was also studied in multipotent mesenchymal stromal cells (MSCs) from human adipose tissue [144]. In a direct cell-cell contact culture system, WJ-MSCs enhanced the expansion of CB-CD34+ cells [145]. Further investigations in this field are required.

## 5. Conclusions

Stem cell therapy is a promising area for future investigations and clinical applications. According to currently available knowledge, it seems to be a safe and effective treatment that can significantly improve functioning in children with CP, although a complete recovery is unlikely. However, due to the severe consequences of this disease and the lack of causal treatment, stem cell therapy remains the main hope for the improvement of these patients’ condition, especially concerning cognitive functions. 

## Figures and Tables

**Table 1:** Neuroprotective properties of growth factors proposed for adjuvant stem cell therapy.

	Cell Targets	Effect
**BDNF**	tTrkB, LNGFR [43], alpha-7 nicotinic receptor [44], reelin [45]	↑ proliferation and differentiation of oligodendrocyte precursor cells [34] ↑ myelination [34]
**EGF**	JAK/STAT, MAPK, Pi3K/Akt [37]	↑ survival, proliferation, and migration of neural precursor cells [37] ↑ differentiation towards oligodendrocyte [37]
		↑ proliferation of astrocytes [37]
**GCSF**	JAK/STAT, MAPK, PI3K/Akt [36]	↑ neurogenesis [46] ↓ apoptosis [46]
**GDNF**	STAT3 [47]	↑ differentiation of neural precursor cells into astrocytes [47,48] ↓ apoptosis [48]
**IGF-1**	ERK, Pi3K/Akt [40]	↑ neural stem cell proliferation and migration [42]

BDNF: Brain-derived neurotrophic factor, EGF: Epidermal growth factor, EGFR: Epidermal growth factor receptor, ERK: Extracellular signal-regulated kinase, GCSF: Granulocyte colony stimulating factor, GDNF: Glial cell line-derived neurotrophic factor, IGF-1: Insulin-like growth factor 1, JAK: Janus kinase, LNGFR: Low-affinity nerve growth factor receptor, MAPK: Mitogen activated protein kinase, PI3K: Phosphoinositide 3-kinase, STAT: Signal transducer and activator of transcription.

**Table 2:** Effects of extracellular vesicle administration in specific diseases.

Disease	Effect
Stroke [59]	↑ synaptogenesis, ↑ angiogenesis
Traumatic brain injury [58]	↓ apoptosis
Inflammation-induced preterm brain injury [57]	↓ inflammation-induced neuronal degeneration, ↓ microgliosis, ↓ astrogliosis
Parkinson’s disease [60]	↓ apoptosis
Alzheimer’s disease [61]	neprilysin secretion
Autism [62]	↓ autistic behaviors
Hepatic ischemia [50]	↓ necrosis, ↓ apoptosis, ↓ inflammation, ↓ oxidative stress
Hepatic failure [51]	↓ apoptosis, ↓ inflammation
Hepatocellular carcinoma [52]	↓ tumor
Myocardial infarction [53]	↑ neovascularization, ↓ inflammation
Myocardial hypoxia [54]	↑ Wnt/β-catenin signaling pathway (↑ survival, ↓ apoptosis)
Ischemic-hypoxic kidney injury [55,56]	↓ apoptosis, ↓ inflammation, ↑ proliferation, ↑ function, ↓ CX3CL1, ↓ CD68+

**Table 3:** Current clinical trials assessing the use of cord blood in children diagnosed with hypoxic-ischemic encephalopathy or cerebral palsy.

ClinicalTrials.gov ID	Therapy	Sample Size (*n*)	Phase	Status on 18 January 2019
NCT02881970	Autologous cord blood	20	I, II	Not yet recruiting
NCT02551003	Autologous cord blood + hypothermia	60	I, II	Recruiting
NCT03352310	Autologous cord blood	40	I	Recruiting
NCT02434965	Autologous cord blood and human placenta-derived stem cells	20	II	Not yet recruiting
NCT02612155	Autologous cord blood	160	II	Recruiting
NCT03123081	Umbilical cord milking	400	NS	Not yet recruiting
NCT03682042	Umbilical cord milking	350	NS	Not yet recruiting
NCT03657394	Umbilical cord milking	1400	III	Not yet recruiting
NCT01072370	Autologous cord blood	40	I, II	Recruiting
NCT03791372	Autologous umbilical cord blood mononuclear cells	25	I	Recruiting
NCT03327467	Autologous or sibling cord blood	NS	NS	Available

NS—not specified.

**Table 4:** Positive and negative effects of quercetin on mesenchymal stem cells.

Parameter	Effect	Model
Proliferation	Increased	Rat BM-MSC cultures [135] Mice BM-MSC cultures [130]
Viability	Increased	Human BM cell cultures [134]
	Decreased	hPSCs [140], hHSC, and progenitors [137], human embryonic NSCs culture [138]
Migration	Increased	Human embryonic NSCs culture [138]
Resistance to oxidative stress	Increased	Human BM cell cultures [134]
Differentiation		
into neural cells	Increased	Human embryonic NSCs culture [138]
into bone cells	Increased	Rat BM-MSCs cultures [129], mice BM-MSC cultures [130]
	Decreased	Human BM cell cultures [133]
into β-cells	Increased	Rat BM-MSCs cultures [131]
into hepatic cells	Increased	Human BM cell cultures [134]
into fat tissue cells	Increased	Human BM cell cultures [133]
Proinflammatory cytokines	Decreased	Rat model of spinal cord injury [132]
Anti-inflammatory cytokines	Increased	Rat model of spinal cord injury [132]
Angiogenic factor secretion	Increased	Rat BM-MSC cultures [135]
BDNF secretion	Increased	Human embryonic NSCs culture [138]

BDNF: Brain-derived neurotrophic factor, BM: Bone marrow, hHSC: Human hematopoietic stem cells, MSC: Mesenchymal stem cells, NSC: Neural stem cells, hPSCs: Human pluripotent stem cells.

## Abbreviations

BDNF	brain-derived neutrophic factor
BM	bone marrow
CB	cord blood
CNS	central nervous system
CP	cerebral palsy
EEG	Electroencephalography
EPC	endothelial progenitor cells
EV	extracellular vesicles
GCSF	granulocyte colony stimulating factor
GMFM-88	Gross Motor Function Measure
HIE	hypoxic-ischemic encephalopathy
HLA	human leukocyte antigen
hUCB	human umbilical cord blood
MDSC	monocyte-derived suppressor cells
MRI	magnetic resonance imaging
MSC	mesenchymal stem cell
OR	odds ratio
TNC	total nucleated cells
Treg	regulatory T cells
TrkB	tropomyosin receptor kinase B
UC	umbilical cord
UCB	umbilical cord blood
WJ	Wharton’s jelly
